# Attrition Within Digital Health Interventions for People With Multiple Sclerosis: Systematic Review and Meta-analysis

**DOI:** 10.2196/27735

**Published:** 2022-02-09

**Authors:** William Bevens, Tracey Weiland, Kathleen Gray, George Jelinek, Sandra Neate, Steve Simpson-Yap

**Affiliations:** 1 Centre for Epidemiology and Biostatistics The University of Melbourne Carlton Australia; 2 Centre for Digital Transformation of Health The University of Melbourne Carlton Australia

**Keywords:** digital health, meta-analysis, self-management, eHealth, attrition, digital health interventions, DHI, multiple sclerosis, MS, randomized controlled trials

## Abstract

**Background:**

Digital health interventions have revolutionized multiple sclerosis (MS) care by supporting people with MS to better self-manage their disease. It is now understood that the technological elements that comprise this category of digital health interventions can influence participant engagement in self-management programs, and people with MS can experience significant barriers, influenced by these elements, to remaining engaged during a period of learning. It is essential to explore the influence of technological elements in mitigating attrition.

**Objective:**

This study aimed to examine the study design and technological elements of documented digital health interventions targeted at people with MS—digital health interventions that were intended to support a program of engagement over a defined period—and to explore how these correlated with attrition among participants of randomized controlled trials (RCTs).

**Methods:**

We conducted a systematic review and meta-analysis of RCTs (n=32) describing digital health self-management interventions for people with MS. We analyzed attrition in included studies, using a random-effects model and meta-regression to measure the association between potential moderators.

**Results:**

There were no measured differences in attrition between the intervention and control arms; however, some of the heterogeneity observed was explained by the composite technological element score. The pooled attrition rates for the intervention and control arms were 14.7% and 15.6%, respectively.

**Conclusions:**

This paper provides insight into the technological composition of digital health interventions designed for people with MS and describes the degree of attrition in both study arms. This paper will aid in the design of future studies in this area, particularly for digital health interventions of this type.

## Introduction

Multiple sclerosis (MS) is a chronic neurological disease that affects over 2 million people globally [1]. The disease course of MS is highly variable and can be associated with a progressive decline in physical and cognitive function. The current treatment for MS involves the use of disease-modifying treatments and symptom management; however, the delivery of health care for MS is becoming increasingly supported by digital health interventions.

People with MS readily seek out web-based information on their disease and appear to do so at rates higher than those with other neurological conditions [2,3]. Further, many people with MS engage with digital technologies to manage their disease, including exchanging medical information with health care providers or to assist in making and maintaining positive lifestyle changes [4]. Digital health interventions that support learning to self-manage MS and scaffold learning and practicing over a specific period of time are a particular subset of digital health interventions for people with MS. Evidence suggests that there are barriers to remaining engaged for the recommended duration in these interventions [5] and technological elements that could support engagement (such as interactivity, multimedia components, or feedback) can play a pivotal role in ameliorating attrition. Understanding the extent to which these technological elements may impact engagement by people with MS in such digital health interventions remains to be explored and is a necessary next step in designing and evaluating such digital health interventions.

We conducted a meta-analysis to address the following primary questions: (1) is there a difference in attrition for participants that were allocated to the intervention in randomized controlled trials (RCTs) of digital health interventions for people with MS between intervention and control arms? (2) How do study characteristics and technological elements of digital health interventions influence observed degrees of attrition?

## Methods

### Eligibility Criteria

Studies were eligible for inclusion in the review if they met each of the following predetermined criteria: (1) describe RCTs examining a treatment intervention or interventions for MS; (2) were delivered via a technological platform (ie, a “computer-assisted” or “web/internet-based intervention” or “eHealth/mHealth”), limited to a PC or to Mac, smartphone, and tablet devices. Interactivity and communication over a specified period of time were important components; therefore, episodic teleconferencing or telemonitoring and virtual reality–only interventions were excluded; (3) included study participants with a diagnosis of MS (any type); (4) reported attrition for those allocated to the intervention or control group upon conclusion of the intervention and study period, and the intervention and control arms were randomized from the same subject population; that is, no comparisons with healthy controls.

### Search Strategy

A literature search was conducted on April 30, 2021, for published studies in the following databases: IEEE, Medline, Scopus, and CINAHL. The search contained terms related to identifying online interventions: (*online* OR *web-based* OR *internet* OR *digital* OR *virtual* OR *computer-assisted* OR *mhealth* OR *mobile* OR *smartphone* OR *ehealth* OR *telehealth* OR *telemedicine* OR *app*), a term identifying multiple sclerosis (*multiple sclerosis*), and a Medical Subject Headings (MeSH) term (*telemedicine*).

Reference lists and in-text citations were also screened for additional studies, as well as recommendations via correspondence from authors of other included studies.

### Selection and Data Collection

**Figure 1:**
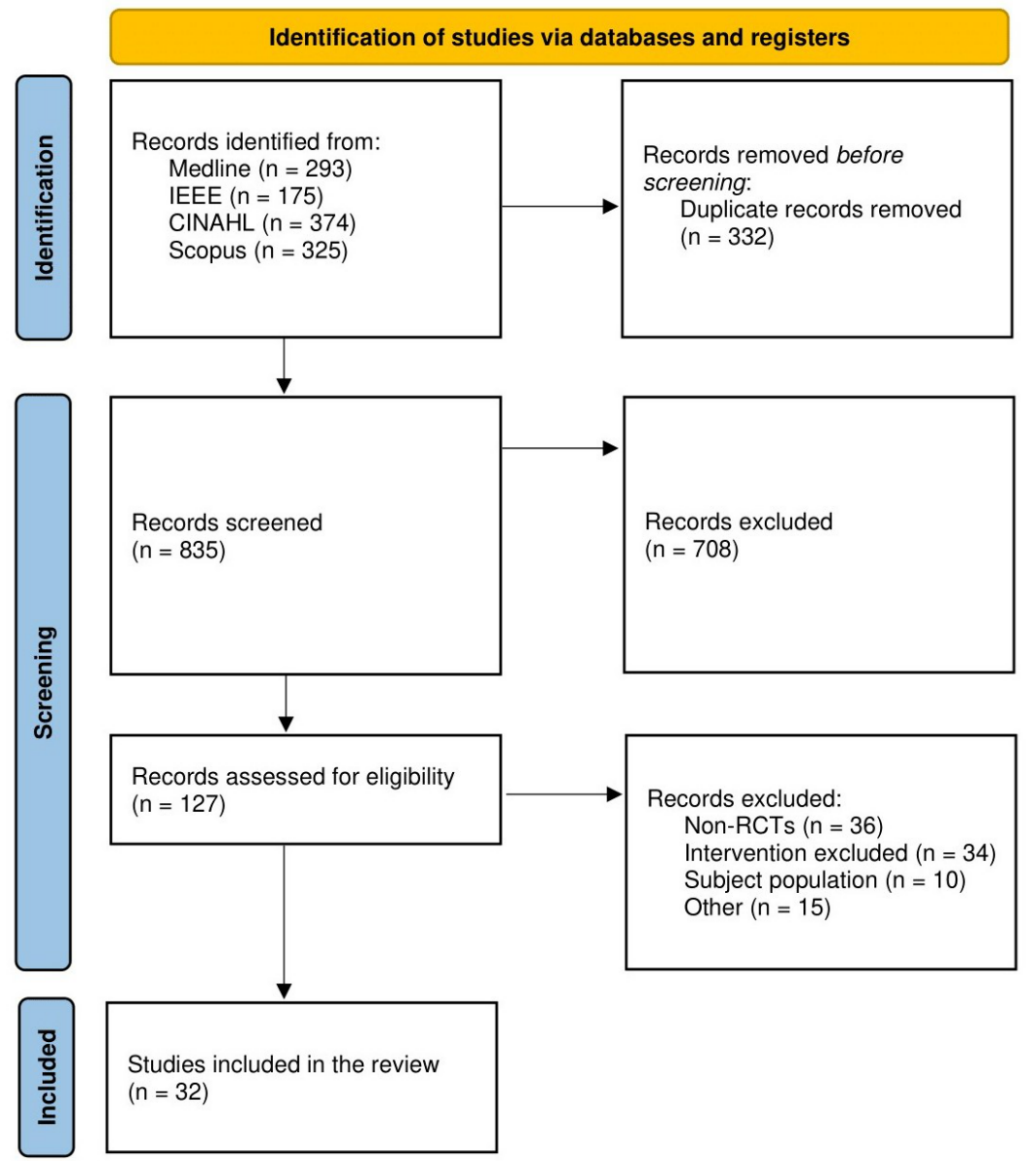
PRISMA (Preferred Reporting Items for Systematic Reviews and Meta-Analyses) flow diagram [6]. RCT: randomized controlled trial.

Duplicates were first removed within and between databases. The remaining articles were then screened on the basis of their titles and abstract; thereafter, the eligibility of the final papers was confirmed following review of the full-text articles (Figure 1). Extraction of study characteristics and outcome data was conducted by authors WB and TW.

The following study characteristics were extracted: study design, study population, country, intervention type, control and comparison intervention, mean age of participants in both arms, percentage of female participants, years since diagnosis, length of intervention in weeks, number of sessions, length of time to final follow-up assessment in weeks, attrition in the intervention and control arms upon conclusion of the intervention, and primary outcome measures. In studies where there were multiple intervention or control arms, attrition data from intervention arms were combined and attrition data from control arms were combined for meta-analysis purposes [7].

Each study was scored on the basis of its technological elements by WB in accordance with the published manuscript. The scoring system used was adapted from Barakat et al [8], who developed it for use in web-based eTherapy programs targeted to eating disorders. Studies were scored in accordance with four domains:

- multimedia channels (written text=“1,” graphics/images=“1,” audio/voiceover=“2,” video=“3,” simulation/3D virtual reality=“4”);
- degree of user interactivity (questionnaires=“1,” quizzes=“1,” goal setting/to‐do list=“2,” homework tasks=“3,” user dashboard=“3,” forums=“3,” self‐monitoring tools=“4,” interactive exercises=“4,” virtual games=“4,” video coaching with professional=“4”);
- level of automated feedback (motivational pop‐ups=“1,” reminders=“2,” nonpersonalized feedback=“3”), personalized feedback (telephone or email)= “4”);
- technological device through which the program was made accessible (outdated technology; eg, compact disk read‐only memory (CD‐ROM)=“1,” modern technology; eg, tablet, desktop computer, laptop, and mobile phone=“2”).

The reporting of technological features of digital health interventions was often vague or absent in publications; therefore, it was necessary to survey each author regarding their examined web-based intervention to clarify scores in accordance with the system adapted from Barakat et al [8]. An email was sent out to authors who had not completed the survey. These authors were sent a summary of the initial scoring by WB and asked to confirm that all the elements listed were present in their study and to identify any that were missed. In total, 19 of the 32 studies responded and the remaining 11 provided additional elements that were not identified in the initial screen by WB. In total, 3 of the 33 studies [9-11] provided access to the program itself and provided confirmation of elements.

### Quality Appraisal

The methodological quality of the final studies included in the review was assessed by author WB using the Cochrane Risk of Bias tool 2 [12]. The tool uses five domains to assess the bias within study design: allocation concealment and random sequence generation, blinding of participants and study personnel, blinding of outcome assessment, incomplete outcome data, and selective reporting of data. Briefly, each domain is composed of questions, to which the response can be “yes,” “possibly yes,” “possibly no,” “no,” and “no information.” Based on answers, each domain is scored as “low risk,” “some concerns,” or “high risk,” whereby the overall judgement is made by the combination of all domains.

### Statistical Analyses

Analyses were conducted using Stata (Stata/SE 16 for Mac, StataCorp).

### Meta-analysis

The Stata command *meta* [13] was used for meta-analysis. The input variables required by *meta* were contained in a 2×2 table; that is, number of individuals who did and did not experience the “outcome event” in either the treatment or the control group. To compare study attrition rates between the intervention groups and the control group, risk ratios were meta-analyzed using the default restricted maximum likelihood method. Log-transformed relative risk of attrition was computed separately for each study, weighted by the inverse of study variance, and pooled to create a summary effect. Studies with no loss to follow-up in one of the intervention or control arms were adjusted by adding 0.5 to each cell within a study’s 2×2 table, enabled by meta-analysis settings within Stata. Studies with no attrition in both intervention and control arms were excluded from the meta-analysis as is best practice [14]. Heterogeneity was assessed using the *I^2^* statistic. Publication bias was assessed by funnel plots. Data were exponentiated and also presented as a Forest plot using the *meta forestplot* command in Stata.

### Meta-regression

Meta-regression was used to examine the relationship between the log-transformed relative attrition rates, and various study characteristics, participant demographics, and intervention technological elements. Study characteristics included the type of control, type of intervention, duration of the study, and duration to the final follow-up. Type of control was dummy-coded as follows: wait-list, active control, usual care or active control computerized (1, 2, 3, 4). The type of intervention was dichotomized owing to small numbers of observations dummy-coded as follows: containing an exercise or physiotherapy condition or other (1, 2). The length of the intervention period was converted to years and evaluated as a continuous variable.

Demographic elements included the type of MS, mean age of the participants, female-to-male ratio, and years since disease onset. The type of MS was dummy-coded as either all MS types or relapsing-remitting MS (1, 2).

The intervention technological elements that could support engagement were as follows: multimedia channels, interactivity, and feedback; overall score was the sum of these 3 elements. This analysis omitted the element that assessed how the intervention was made accessible since all interventions used desktop computers. An attempt was made to further clarify whether interventions were designed to accommodate multiple platforms, but this remained unclear for many studies.

Variables were first examined individually and then jointly as a single meta-regression model informed by a directed acyclic graph.

## Results

### Results Overview

The database search retrieved 1167 articles, of which 187 were duplicates. After duplicates were removed, titles and abstracts of the remaining 835 were screened. Of these, 127 articles were obtained for full-text review. In total, 95 papers did not meet the inclusion criteria and were excluded after full-text review. Of the excluded papers, 34 were not applicable interventions, 36 were not RCTs, 10 had the wrong subject population, and 15 others were excluded for other reasons (Multimedia Appendix 1). In total, 32 papers that described 32 studies were eligible for meta-analysis.

### Study Characteristics

**Figure 2:**
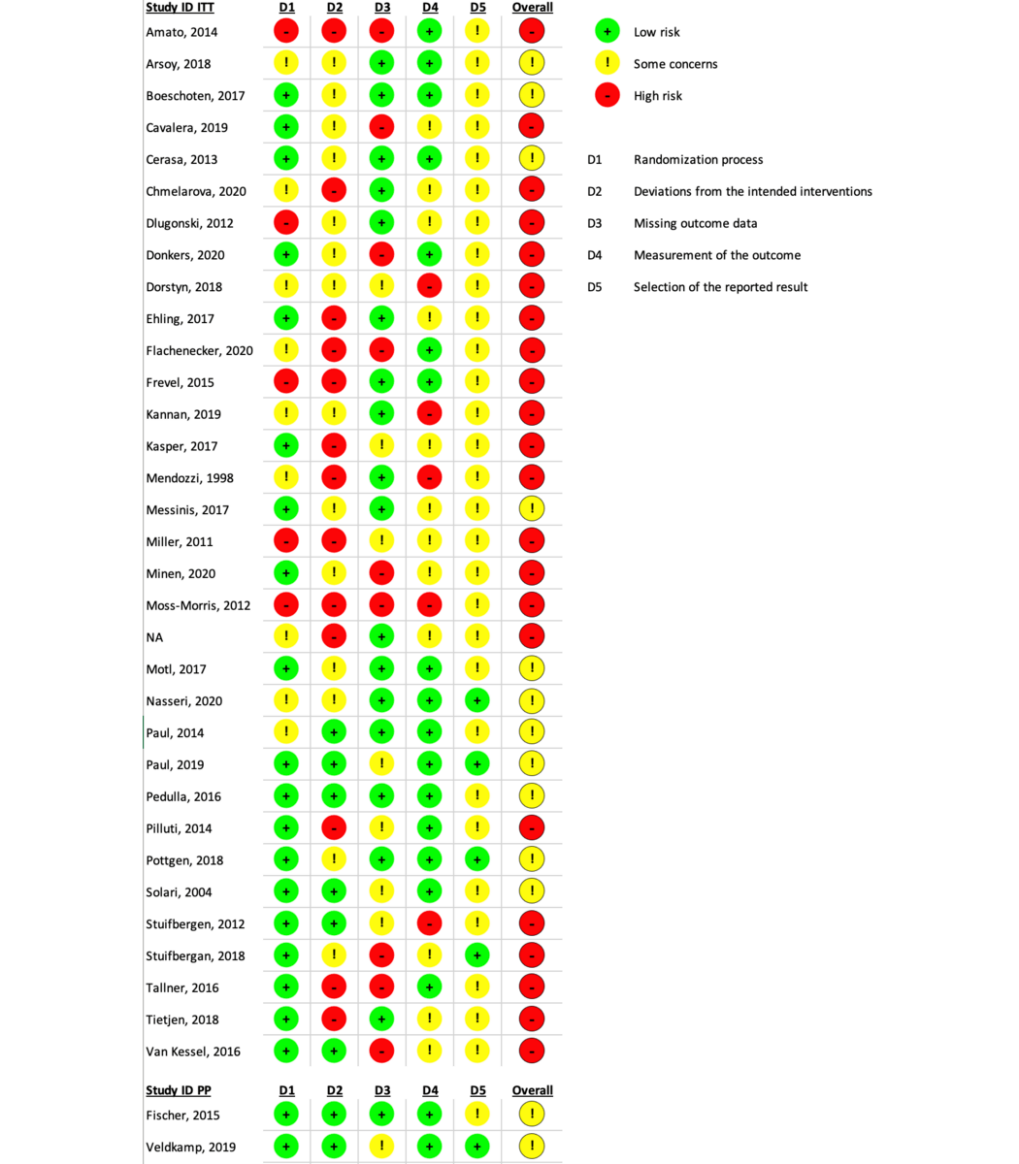
Risk of bias assessment summary using the Cochrane Risk of Bias tool. ITT: intention-to-treat; PP: per-protocol.

Details of the study characteristics for the 32 included papers are reported in Table 1. Of the 32 studies, 10 were published before 2015, and the earliest paper was published in 2004. A total of 23 of 32 studies included all types of MS, 8 included only people with relapsing-remitting MS, and 1 included primary-progressive MS (PPMS). Two included any type of MS if fatigue was a symptom, 2 others included any type of MS if depression was a symptom, 1 assessed whether participants experienced migraines, and 1 only assessed whether participants experienced severe disability. The mean length of interventions was 14.0 (SD 10.2) weeks, and the mean length to last follow-up was 20.9 (SD 12.1) weeks. In total, 20 of 32 studies reported a high risk of bias upon quality appraisal with none reporting low risk (Figure 2).

**Table 1:** Characteristics of the included studies.

Study	Population	Country	Participant age (years), mean	Females, %	Intervention type	Control type	Length of the intervention (weeks)	Maximum follow-up (weeks)
Dlugonski, 2012 [15]	Relapsing-remitting multiple sclerosis	United States	46.65	84	Exercise	Wait-list	12	13
Donkers, 2020 [16]	All types of multiple sclerosis	Canada	41.30	85	Exercise	Wait-list	4	4
Ehling, 2017 [17]	All types of multiple sclerosis	Austria	48.60	45	Exercise	Active	24	26
Flachenecker, 2020 [18]	All types of multiple sclerosis	Germany	47.00	61	Exercise	Usual care	13	39
Frevel, 2015 [19]	Relapsing-remitting multiple sclerosis	Germany	45.60	84	Exercise and falls prevention training	Active	12	12
Kannan, 2019 [20]	All types of multiple sclerosis	United States	55.75	70	Exercise	Wait-list	8	22
Motl, 2011 [21]	Relapsing-remitting multiple sclerosis	United States	45.85	91	Exercise	Wait-list	13	13
Motl, 2017 [22]	All types of multiple sclerosis	United States	51.90	85	Exercise	Wait-list	24	24
Nasseri, 2020 [23]	Primary-progressive multiple sclerosis	Germany	51.10	51	Exercise	Usual care	12	12
Pilluti, 2014 [24]	All types of multiple sclerosis	United States	49.00	76	Exercise	Active (computer)	8	26
Tallner, 2016 [25]	All types of multiple sclerosis	Germany	40.80	75	Exercise	Wait-list	13	26
Paul, 2014 [10]	All types of multiple sclerosis	United Kingdom	51.25	75	Physiotherapy	Wait-list	12	13
Paul, 2019 [11]	All types of multiple sclerosis	United Kingdom	56.05	77	Physiotherapy	Active	26	39
Amato, 2014 [26]	Relapsing-remitting multiple sclerosis	Italy	40.9	78	Cognitive rehabilitation	Active (computer)	36	49
Boeschoten, 2017 [27]	All types of multiple sclerosis with depression	Netherlands	48.90	82	Problem-solving treatment for depression	Wait-list	10	17.4
Chmelařová, 2020 [28]	All types of multiple sclerosis	Czech Republic	41.90	78	Cognitive rehabilitation	Active	8	8
Fischer, 2015 [29]	All types of multiple sclerosis with depression	Germany	45.28	78	Cognitive behavioural therapy	Wait-list	9	26
Messinis, 2017 [30]	Relapsing-remitting multiple sclerosis	Greece	45.60	69	Cognitive rehabilitation	Usual care	10	26
Minen, 2020 [31]	All types of multiple sclerosis with migraine	United States	39.70	89	Cognitive behavioural therapy	Usual care	13	26
Moss-Morris, 2012 [32]	All types of multiple sclerosis	United Kingdom	40.95	0.82	Cognitive behavioural therapy	Wait-list	8	10
Pedulla, 2016 [33]	All types of multiple sclerosis	Italy	47.60	68	Cognitive training	Active (computer)	8	26
Pottgen, 2018 [34]	All types of multiple sclerosis with fatigue	Germany	41.35	81	Cognitive behavioural therapy	Wait-list	12	24
Solari, 2004 [35]	All types of multiple sclerosis	Italy	43.70	64	Cognitive behavioural therapy	Active (computer)	8	16
Stuifbergen 2012 [36]	All types of multiple sclerosis	United States	N/A^a^	89	Group cognitive rehabilitation	Wait-list	8	13
Stuifbergen 2018 [37]	All types of multiple sclerosis	United States	N/A	88	Group cognitive rehabilitation	Usual care	8	26
van Kessel, 2016 [38]	All types of multiple sclerosis with fatigue	New Zealand	43.00	74	Cognitive behavioural therapy	Active	10	10
Veldkamp, 2019 [39]	All types of multiple sclerosis	Belgium	52.4	58	Cognitive training	Active	8	12
Cavalera, 2019 [40]	Relapsing-remitting multiple sclerosis	Italy	42.73	65	Mindfulness training	Active	8	26
Kasper, 2017 [41]	All types of multiple sclerosis	Germany	40.1	71	Health literacy	Active (computer)	0.14	0.14
Miller, 2011 [42]	All types of multiple sclerosis	United States	48.10	79	Falls prevention training	Active	52	52
Dorstyn, 2018 [9]	Relapsing-remitting multiple sclerosis	Australia	41.30	85	PowerPoint to build work skills	Wait-list	4	4
Cerasa, 2013 [43]	Relapsing-remitting multiple sclerosis	Italy	32.7	75	Cognitive rehabilitation	Active (computer)	6	6

^a^N/A: not applicable.

### Study Populations

The mean sample size across all 32 studies was 97.7 (SD 121.8). The smallest study contained 18 participants [19] and the largest 682 participants [41]. The median numbers of participants in the intervention and control arms at the final follow-up were 33 and 27, respectively.

### Types of Digital Health Intervention and Control

All studies included a program aimed at people with MS delivered via web-based technology, with 2 studies [36,37] that included in-person components. The program ranged from 0.14 week to 52 weeks intended duration. In total, 15 of the 32 studies included a cognitive behavioral training element, 11 included an exercise training component, 2 included web-based physiotherapy, 1 included a fall prevention intervention, 1 included a program to build “returning to work” skills, 1 included a program to build health literacy, and 1 included a mindfulness intervention.

In total, 12 of the 32 studies used a wait-list control, while 7 studies used a usual care control. Eight studies used a noncomputerized active control, which included a physical activity regime [17-19,42], physiotherapy [11], modified version of the intervention [38,39], or non–MS-specific psychoeducation [40]. Five studies used a computerized active control, which were all broadly modified versions of the intervention [26,33,35,41,43].

### Digital Health Intervention Characteristics App

Complete scoring information for each individual study is available in Multimedia Appendix 2.

### Multimedia

The mean multimedia score across all 32 studies was 3.5 (SD 2.2). In total, 29 of the 32 studies contained written text. Two of the remaining 3 studies that did not include written text were a gamified web-based cognitive training program [36,37], and 1 study described a web-based, asynchronous messaging system that uses electronic personal health records [42]. In total, 15 of 32 studies contained video or animation, half of all studies including images or graphics, and only one study used audio voiceover to augment written material [27].

### Interactivity

The mean interactivity score across all 32 studies was 5.2 (SD 3.3). Eight of 32 studies contained homework tasks [9,27,32,34, 36-38,40], which varied from set questions or self-directed learning to diary entries between course sessions. In total, 15 of 33 studies contained a self-monitoring component [10,11,15,16,18,21-25,31,32,38,42], which varied from a validated tool integrated within the program to generic mood-related questions and diary entries. Nine studies included interactive exercises or games [26,28,30,33,35-37,39,43], 5 studies contained goal-setting elements [15,21,22,24,38], 3 contained forums for interaction with other participants [20-22], 3 contained video coaching or interactive meditation by professionals [15,24,40], and 1 contained a questionnaire or quiz [32].

### Feedback

The mean feedback score across all 32 studies was 2.8 (SD 2.4). In total, 14 of 32 studies included feedback [9-11,16, 18,19,21,22,27,28,36,37,39,40], which ranged from personalized feedback from a trained staff member or professional to general feedback delivered automatically via the web-based platform. Reminders were included in 6 of 17 studies [15,17,20,25,32,42], which prompted users to complete the sessions, while 2 studies also included reminders on when new content was added or existing content was updated [21,22].

### Meta-analysis

**Figure 3:**
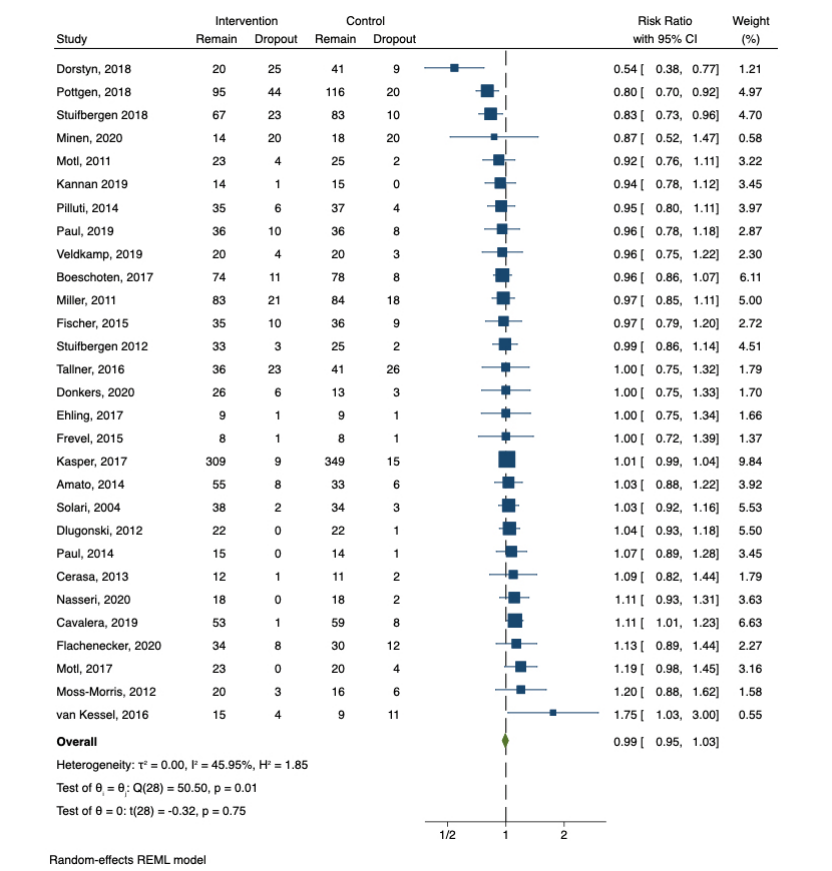
Individual effect sizes and forest plot of the difference in the degree of attrition between the intervention and control arms of the included studies within our analysis (negative values indicate greater attrition in the intervention arm than in the control arm and vice versa).

**Figure 4:**
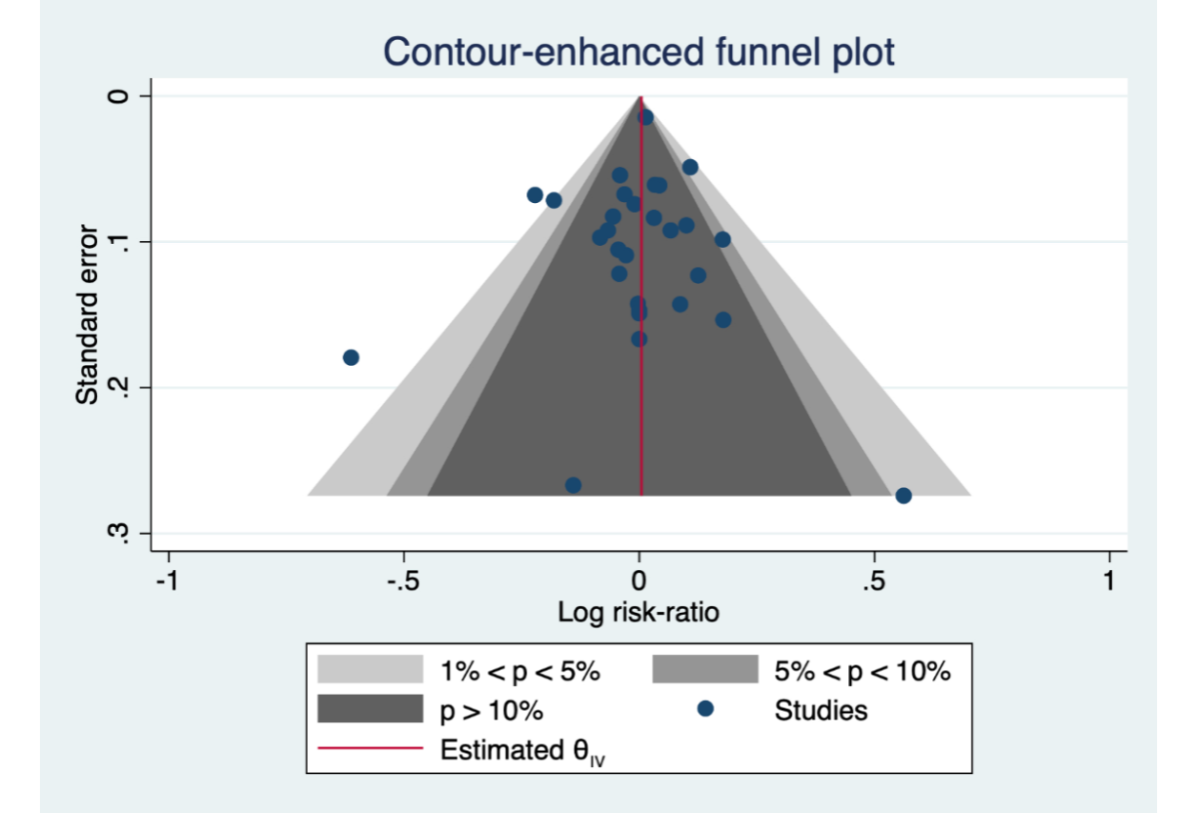
Contour-enhanced funnel plot of the relative attrition rates (>0 indicates a higher attrition rate in the intervention condition).

The final meta-analysis included 29 studies after 3 exclusions due to no attrition in both the intervention and control arms. Meta-analysis described an overall effect size of =–0.993 (95% CI 0.95-1.04) and a *P* value of .75, which indicates no statistical difference in the degree of attrition between the intervention and control arms (Figure 3). The test of homogeneity of study-specific effect sizes (*Q*) was rejected (*P*<.01). The data also suggested the presence of a medium level of heterogeneity between studies (*I^2^=*50.50). There is evidence of publication bias as described by the funnel plot showing some asymmetry (Figure 4).

### Meta-regression

#### Univariable Association

Univariable assessment of study characteristics and eHealth elements are described in Table 2. No significant association between length-of-treatment, weeks to last follow-up, type of control, MS type, feedback or interactivity, or attrition was observed. Lower attrition was observed when eHealth elements overall score and multimedia score were higher.

**Table 2:** Univariable meta-regression model describing the association between differential attrition and study characteristics.

Variables	Coefficient (95% CI)	*P* value
Length of treatment (years)	1.00 (1.00-1.00)	.88
Active control	1.03 (0.92-1.15)	.64
Usual care	1.07 (0.92-1.23)	.38
Active control computerized	1.06 (0.94-1.20)	.29
Relapsing-remitting multiple sclerosis	1.03 (0.93-1.14)	.60
Primary-progressive multiple sclerosis	1.12 (0.89-1.42)	.31
Non–cognitive behavioral therapy intervention	1.01 (0.92-1.10)	.88
Mean age (years)	1.00 (0.99-1.01)	.60
Female-to-male ratio	0.99 (0.99-1.00)	.01
Years since onset	1.01 (0.99-1.04)	.28
Overall score	1.00 (1.00-1.01)	.20
Multimedia subscore	*1.02* (*1.00-1.04*)^a^	*.03*
Interactivity subscore	1.00 (0.99-1.02)	.47
Feedback subscore	1.00 (0.98-1.02)	.79

^a^Italicized values are significant at *P*<.05.

#### Multivariable Models

Adjustment for other covariates did materially impact the association of overall score with attrition, which was significant (*P*<.01; Table 3). After adjustment, the multimedia subscore remained a significant association (*P*=.03; Table 4).

**Table 3:** Multivariable meta-regression model describing the association between overall score and attrition.

Variables	Risk ratio (95% CI)	*P* value
Overall score	*1.01* (*1.00-1.03*)^a^	*.01*
Length of treatment (years)	1.08 (0.85-1.39)	.50
Active control	1.04 (0.88-1.22)	.46
Usual care	0.96 (0.79-1.16)	.64
Active control computerized	1.15 (0.97-1.37)	.09
Nonexercise intervention	0.97 (0.88-1.08)	.59
Relapsing-remitting multiple sclerosis	0.99 (0.88-1.12)	.84
Primary-progressive multiple sclerosis	1.11 (0.82-1.49)	.48
Female-to-male ratio	0.99 (0.99-1.00)	.13
Mean age (years)	1.00 (0.99-1.01)	.91

^a^Italicized values are significant at *P*<.05.

**Table 4:** Multivariable meta-regression model describing the association between multimedia subscore and attrition.

Variables	Risk ratio (95% CI)	*P* value
Multimedia subscore	*1.04 (1.00-1.08)* ^a^	*.03*
Length of treatment (years)	1.14 (0.87-1.48)	.32
Active control	1.05 (0.89-1.24)	.53
Usual care	0.99 (0.82-1.20)	.94
Active control computerized	1.09 (0.93-1.28)	.28
Nonexercise intervention	1.01 (0.92-1.11)	.84
Relapsing-remitting multiple sclerosis	1.01 (0.90-1.14)	.80
Primary-progressive multiple sclerosis	1.06 (0.79-1.42)	.68
Female-to-male ratio	1.00 (0.99-1.00)	.34
Mean age (years)	1.00 (0.98-1.01)	.55

^a^Italicized values are significant at *P*<.05.

## Discussion

### Principal Findings

The aim of this systematic review and meta-analysis was to describe the study characteristics and technological components of digital health interventions that supported people with MS through a program of learning and practicing self-management, and then investigate any associations these factors may have with attrition. Our meta-analysis found no difference in attrition between participants allocated to intervention and control arms of the included RCTs. A multivariable association was found between degree of attrition and overall score, which was not observed in the univariable model, and between the degree of attrition and the multimedia subscore in both univariable and multivariable models. This suggests that among included studies, the interactivity subscore was the main driver in the association observed between overall score and degree of attrition; that is, the likelihood of attrition within the intervention arm compared with the control arm was associated with the inclusion of interactive elements such as self-directed tasks or self-monitoring.

Only 8 of the 32 studies described a design process or framework by which the intervention was developed. Development methods included engagement with the MS community [9,10,31,32,38], design with practitioners and allied health professionals [34], both [31], or followed a preset development plan [23]. There was no association observed between the number or type of technological elements used within the intervention and whether the study used or described a development framework. It is possible that a greater degree of consultation with the MS community during design phases does not necessarily lead to a great quantity of or more complex technological features. This highlights that while engagement with communities during the design phase is important, it is crucial to ensure this engagement is constructive and not perfunctory. Population preferences for number and type of features require further exploration; however, our findings suggest that elaborate features are not necessarily what people with MS want in a web-based intervention of this kind.

Interestingly, length of intervention was not associated with attrition in either control or intervention arms. Intuitively, the longer the intervention itself, the greater the potential attrition from these studies, in which a negative relationship between attrition and length of treatment for self-management interventions for people with MS has been reported [45]. It is possible that the role of treatment length in attrition may only be true at longer time frames than in the included studies of both meta-analyses. Another likely variable is the number of sessions or time in contact with participants. It is possible that rather than the length of the intervention being a mediator, it is the time participants are exposed to the intervention (“dose”). Studies infrequently report these data; therefore, this needs to be addressed in future studies.

Another interesting finding is that the mean average attrition rates in the intervention and control arms were 14.7% and 15.6%, respectively, which is a useful figure for calculating sample sizes for future studies. A previous meta-analysis investigating attrition rates based on published studies of existing self-management interventions for people with MS reported pooled attrition rates of 16.8% and 14.4% for the intervention and control groups, respectively [45]. Interestingly, studies that used face-to-face delivery of interventions were described as having higher attrition rates. Our analysis supports their pooled data attrition rates, which indicates that there may not actually be a difference between face-to-face and non–face-to-face digital health interventions of this kind. Further, their meta-analysis described associations between attrition and sex, age, and length of intervention, which was not observed in our data set. As their data was pooled between face-to-face and other modes of delivery, it is possible that the web-based mode of delivery may be mediating that relationship in our analysis.

Importantly, 9 of 32 studies did not report any outcomes related to the use of the intervention or technological elements within their intervention; they only reported primary outcome measures related to health outcomes. In total, 15 of 32 studies reported logins, app use, or sessions completed, while only 4 studies reported any interactions between participants and technological elements that were not directly related to the primary outcome measure. This has implications of measuring the “dose” for digital health interventions, whereby factors not reported may have an impact on the outcome measures reported as well as on attrition. Studies describing digital health interventions should address this in the future.

### Limitations

Technological elements were underreported in published papers as evidenced by the authors of 10 of 32 studies needing to be followed up to provide information not accessible within the papers. It is possible that this analysis failed to capture the entirety of the included technological elements present within these studies; however, this is unlikely with the follow-up procedure. The need for better reporting of digital health interventions is crucial in ensuring reproducibility and assessment of web-based interventions, especially as several accessible frameworks exist directed at digital tools [44,46] or more generally [47]. Providing access to explore the intervention itself—as 3 authors did for this study—may be another solution, although it is not feasible for some studies.

Digital health interventions are not new; however, there have been fewer published studies on their use in MS compared with other chronic conditions; a greater number of studies would make the meta-regression more reliable. Further stratification of participants may provide greater insight into the attrition in these interventions by accounting for digital literacy, level of access to technology infrastructure, and better general reporting of the intervention design overall.

While the scoring system used for technological component ratings in this review has previously been used and was created in accordance with guidelines for digital intervention development, this system is not yet validated. Several additions were made to this scoring system to accommodate features that were not included, and it is possible that these additions have compromised internal validity. Analysis was undertaken using the previous, unmodified scoring system and revealed no changes to the results using our modified scoring system; however, caution must be applied to interpreting these results before validation has occurred. Additionally, while the scoring was derived from both publication and authors themselves, it is possible there were inaccuracies due to misinterpretation or inability to recall.

It is possible that using qualitative methods and direct observations to understand the ways in which people with MS engage with technological components of these digital health interventions is a better predictor of attrition than merely quantifying and scoring the components themselves. Unfortunately, no studies provided the data required to carry out these analyses. It also possible that analyzing individual components of the subscores for multimedia, interactivity and feedback, rather than overall subscores themselves, may elucidate further associations; however, the weighting within these subscores of the different components aims to address this.

The inclusion criteria were also strict on the definition of the digital health interventions of interest to us. This excluded studies that were exclusively virtual reality interventions and those that were exclusively telemonitoring or conferencing. These exclusions may have biased our data. It is noteworthy that the category of digital health interventions of interest to us is not well-defined in the major taxonomy of digital health interventions published by the World Health Organization; the category *Interventions for Clients*, this describes “communication” but overlooks the structuring and scaffolding of interventions that would be better described as “education” [48].

### Conclusions

In conclusion, this study describes no difference in the rates of attrition between participants allocated to the intervention and control arms in digital health interventions for people with MS. An association between overall technological elements score and attrition was observed; however, the underlying mechanism is unclear. Future studies should further investigate the roles these elements play in retention in web-based interventions.

## Appendix

Multimedia Appendix 1 Numbers of inclusions/exclusions at each stage of study selection, including reasons for exclusion.

Multimedia Appendix 2 Rubric for scoring of individual studies and their technological elements.

## Abbreviations

CD-ROM: compact disk read‐only memory
MeSH: Medical Subject Headings
MS: multiple sclerosis
PPMS: primary-progressive multiple sclerosis
RCT: randomized controlled trial
