# Supplementary material for: Attrition Within Digital Health Interventions for People With Multiple Sclerosis: Systematic Review and Meta-analysis
Source: J Med Internet Res. 2022 Feb 9;24(2):e27735. doi: 10.2196/27735 (PMC8867299; doi:10.2196/27735)
Supplement: Multimedia Appendix 1 [file jmir_v24i2e27735_app1.docx]

| Database | Search terms – 30/03/2021 | Results |
| --- | --- | --- |
| IEEE | ((("Publication Title": online OR web-based OR internet OR digital OR virtual OR computer-assisted OR mhealth OR mobile OR smartphone OR ehealth OR telehealth OR telemedicine OR app) AND "multiple sclerosis") OR (("Mesh_Terms": "telemedicine") AND multiple sclerosis) | 175 |
| Medline | ((((online or web-based or internet or digital or virtual or computer-assisted or mhealth or mobile or smartphone or ehealth or telehealth or telemedicine or app) and multiple sclerosis).ti. not (review or protocol).pt.) or telemedicine.sh.) and multiple sclerosis.ti. | 293 |
| Cinahl | TI(online OR web-based OR internet OR digital OR virtual OR computer-assisted OR mhealth OR mobile OR smartphone OR ehealth OR telehealth OR telemedicine OR app) AND TI(multiple sclerosis) OR MESH(telemedicine) AND TI(multiple sclerosis) | 374 |
| Scopus | TITLE ( *online*  OR  *web-based*  OR  *internet*  OR  *digital*  OR  *virtual*  OR  *computer-assisted*  OR  *mhealth*  OR  *mobile*  OR  *smartphone*  OR  *ehealth*  OR  *telehealth*  OR  *telemedicine*  OR  *app* )  AND  TITLE-ABS ( *"multiple sclerosis"* )  DOCTYPE ( *ar*  AND NOT  *re* ) | 325 |
| **Total** |  | **1167** |
|  | Duplicates | 332 |
|  | Remaining | **835** |
| **Title/abs** |  |  |
|  | Subject population wrong/unclear | 45 |
|  | Review | 28 |
|  | Conference/Meeting (not enough data) | 1 |
|  | Monitoring/Diagnostic | 80 |
|  | No author information in citation |  |
|  | Survey | 25 |
|  | Protocol | 46 |
|  | Non-control trial | 234 |
|  | Statement of retraction/correction | 3 |
|  | Secondary analysis |  |
|  | Incorrect intervention: pharmacological/telephone/non-digital/teleconsultation | 244 |
|  | Incomplete citation | 2 |
| **Total** |  |  |
|  | Excluded | 708 |
|  | Remaining | 127 |
| **Full text** |  |  |
|  | Intervention not applicable: telephone delivered, non-computer/tablet/smartphone/virtual reality | 34 |
|  | Non-RCT | 36 |
|  | Subject population wrong/not identified | 9 |
|  | Secondary analysis | 1 |
|  | Self-monitoring; no intervention | 9 |
|  | Abstract in conference – not enough data | 1 |
|  | Landtblom, 2019 – interesting as no centralised system; disparate texts, emails and phone calls. | 1 |
|  | Published a correction to existing article | 1 |
|  | Wrong control population (healthy control) | 3 |
| **Final** |  |  |
|  | Excluded | 95 |
|  | Included (one new study added in from reference list in other included study!). | 32 |
