# Supplementary material for: Attrition Within Digital Health Interventions for People With Multiple Sclerosis: Systematic Review and Meta-analysis
Source: J Med Internet Res. 2022 Feb 9;24(2):e27735. doi: 10.2196/27735 (PMC8867299; doi:10.2196/27735)
Supplement: Multimedia Appendix 2 [file jmir_v24i2e27735_app2.docx]

*multimedia channels* employed (written text = “1,” graphics/images = “1,” audio/voice‐over = “2,” video = “3,” simulation/3D virtual reality = “4”); degree of user *interactivity* (questionnaires = “1,” quizzes = “1,” goal setting/to‐do list = “2,” homework tasks = “3,” user dashboard = “3,” forums = “3,” self‐monitoring tools = “4,” interactive exercises = “4,” virtual games = “4,” Video coaching with professional = “4”); level of *automated feedback* (motivational pop‐ups = “1,” reminders = “2,” nonpersonalized feedback = “3”; personalized feedback (telephone and email)= “4”); and *technological device* through which the program was made accessible (outdated technology for example, compact disk, read‐only‐memory (CD‐ROM) = “1,” modern technology for example, tablet, personal computer (PC), laptop, phone = “2”).

Additional “1” point for audio/voice‐overs and additional “2” points for video or simulation/3D virtual reality). An additional “1” point was given to studies where it was indicated that automated feedback/support was provided more than twice per week.

*Red text indicates scoring that has been introduced in addition to the original methodology.*

|  | Multimedia Channels | Interactivity | Automated Feedback | Platform | ***Score*** |
| --- | --- | --- | --- | --- | --- |
| Amato, 2014 | Text (1) Images (1) | Interactive exercises/games (4) |  | PC | 6 |
| Boeschoten 2017 | Text (1) Images (1) voice-over (2) | Homework tasks (3)  Support from trainers – no limit (1) self-monitoring tools (4) user dashboard (4) | Reminders (2) Personalized feedback (4) | PC | 22 |
| Cavalera 2019 | Video (3) Written text (1) | Interactive exercises/meditation via skype (4) homework tasks (3) | Personalized feedback (4) | PC | 15 |
| Cerasa, 2013 | Text (1) Images (1) Video (3) | Interactive exercises/games (4) |  | PC | 9 |
| Chmelarova, 2020 | Text (1) Images (1) Video (3) | Interactive exercises/games (4), user dashboard (3) | Automated feedback (3) | PC | 15 |
| Dlugonski 2012 | Video (3) Text (1) | Video v with professional (4) self-monitoring tools (4) goal setting (3) | Reminders (2) | PC | 17 |
| Donkers, 2020 | Text (1) Video (3) Audio (2) | Self-monitoring (4) | Personalized feedback (4) | PC/Mac | 14 |
| Dorstyn 2018 [Powerpoint] | Text (1) Images (1) | Homework tasks (3) | Personalized feedback (4) | PC | 11.5 |
| Ehling, 2017 | Text (1) Video (3) |  | Reminders (2) | Tablet | 6 |
| Fischer 2015 | Text (1) Images (1) Animation (3) |  |  | PC | 5 |
| Flachenecker, 2020 | Text (1) Images (1) | Self-monitoring (4) | Personalised feedback, telephone, instant messaging, email and video (4) video coaching with professional (4) | PC | 14 |
| Frevel 2015 | Text (1) |  | Personalised feedback from staff (4) | PC | 5 |
| Kannan 2019 | Text (1) Images (1) Video (3) audio (2) | Forums (3) | Reminders (2) | PC | 12 |
| Kasper, 2017 | Text (1) Images (1) |  |  | PC | 2 |
| Messinis, 2017 | Text (1) Images (1) | Interactive exercises/games (4) |  | PC | 6 |
| Miller 2011 |  | Self-monitoring (4) | Reminders (2) | PC | 8.5 |
| Minen, 2020 | Text (1) Images (1) Audio (2) | Self-monitoring (4) |  | Smartphone | 8 |
| Moss-Morris 2012 | Text (1) Images (1) Video (3) Audio (2) | Self-monitoring (4) self-directed homework tasks (3) questionnaire (1) goal-setting (2) | Synchornous telephone support from staff 3x 30-60mins during modules (4) reminders (2) | PC | 23 |
| Motl, 2011 | Text (1) Video (3) | Goal setting (2) Forum (3) self-monitoring (4) | Updates on new content/reminders (2) personalized feedback (4) | PC | 19 |
| Motl, 2017 | Text (1) Video (3) Audio (2) | Goal setting (2) Forum (3) self-monitoring (4) | Updates on new content/reminders (2) personalized feedback (4) Video support/expert support (4) | PC | 17 |
| Nasseri, 2020 | Video (3) Images (1) | Self-monitoring (4) |  | Smartphone | 8 |
| Paul 2014 | Text (1) Video (3) Audio (2) | Self-monitoring (4) | Feedback (4) | PC | 14 |
| Paul 2019 | Text (1) Video (3) Audio (2) | Self-monitoring (4) | Feedback (4) | PC | 14 |
| Pedulla, 2016 |  | Interactive exercises/games (4) |  | Smartphone | 4 |
| Pilutti, 2014 | Video (3) Text (1) | Video coaching with professional (4) self-monitoring tools (4) goal setting (3) | Reminders (2) | PC | 17 |
| Pottgen 2018 | Text (1) | homework (3) |  | PC | 4 |
| Solari, 2004 | Text (1) Images (1) | Interactive exercises/games (4) |  | PC | 6 |
| Stuifbergan 2012 |  | Interactive exercises/games (4) homework tasks (3) | Feedback (4) | PC | 11 |
| Stuifbergan 2018 |  | Interactive exercises/games (4) homework tasks (3) | Feedback (4) | PC | 11 |
| Tallner 2016 | Text (1) Images (1) | Self-monitoring (4) | Staff support, asynchronous, online messaging, rarely email and telephone (4) reminders (2) | PC | 12 |
|  |  |  |  |  |  |
| Van Kessel 2016 | Text (1) Images (1) Video (3) Audio (2) | Self-monitoring (4) homework tasks (3) goal setting (2) questionnaire (1) | Telephone support from staff 3x 30-60mins (4) reminders (2) | PC | 23 |
| Veldkamp, 2019 | Audio (2) Text (1) | Interactive exercises/games(4) | automated feedback (3) | PC | 10 |

 changed due to contact

 response for this paper

 accessed the intervention personally
